# The modulatory effect of high salt on immune cells and related diseases

**DOI:** 10.1111/cpr.13250

**Published:** 2022-06-23

**Authors:** Xian Li, Aqu Alu, Yuquan Wei, Xiawei Wei, Min Luo

**Affiliations:** ^1^ Laboratory of Aging Research and Cancer Drug Target, State Key Laboratory of Biotherapy and Cancer Center, National Clinical Research Center for Geriatrics, West China Hospital Sichuan University Chengdu China

## Abstract

**Background:**

The adverse effect of excessive salt intake has been recognized in decades. Researchers have mainly focused on the association between salt intake and hypertension. However, studies in recent years have proposed the existence of extra‐renal sodium storage and provided insight into the immunomodulatory function of sodium.

**Objectives:**

In this review, we discuss the modulatory effects of high salt on various innate and adaptive immune cells and immune‐regulated diseases.

**Methods:**

We identified papers through electronic searches of PubMed database from inception to March 2022.

**Results:**

An increasing body of evidence has demonstrated that high salt can modulate the differentiation, activation and function of multiple immune cells. Furthermore, a high‐salt diet can increase tissue sodium concentrations and influence the immune responses in microenvironments, thereby affecting the development of immune‐regulated diseases, including hypertension, multiple sclerosis, cancer and infections. These findings provide a novel mechanism for the pathology of certain diseases and indicate that salt might serve as a target or potential therapeutic agent in different disease contexts.

**Conclusion:**

High salt has a profound impact on the differentiation, activation and function of multiple immune cells. Additionally, an HSD can modulate the development of various immune‐regulated diseases.

## Graphical abstract

This review describes the modulatory effect of high salt on various immune cells and immune‐regulated diseases. High salt significantly affects the differentiation, activation and function of multiple innate and adaptive immune cells and dominantly induces a pro‐inflammatory state in microenvironments, thus influencing the development of various immune‐regulated diseases and serving as a target or potential therapeutic agent in different disease contexts.

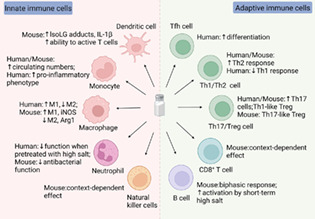

## 1 INTRODUCTION

Salt (NaCl), common in our daily life, can be found naturally in various foods and is used in food manufacturing, the chemical industry, clinical therapy and so forth. For instance, salty condiments contain plenty of NaCl, whereas saline is the most frequently administered intravenous fluid.^1^ Although salt is necessary for the human body, excessive salt intake can be detrimental and increase the risk of diseases such as hypertension, heart failure and renal disease.^2^ In addition, the immunomodulatory function of salt has been reported. Early studies found that high salt increased the cytokine production in peripheral blood mononuclear cells (PBMCs).^3^, ^4^ In the past decades, growing evidence has indicated that high salt can influence various immune cells. Moreover, a high‐salt diet (HSD) has a pronounced effect on immune‐regulated diseases, and salt is potentially applied in immune therapy. This review will summarize some of the recent advances in the immunomodulatory effect of high salt. We will first introduce sodium homeostasis and its physiological functions. Then, we focused on the effect of high salt on various immune cells in microenvironments. Furthermore, the influence of HSD on immune‐regulated diseases and relevant immune responses will be reviewed. Finally, we will briefly discuss the present and potential clinical applications of high salt.

## 2 SODIUM HOMEOSTASIS AND PHYSIOLOGICAL FUNCTIONS

Dietary salt is an important source of sodium, of which homeostasis is tightly regulated.^5^ Sodium homeostasis in the traditional two‐compartment model is regulated by the kidney.^6^ However, in recent years, studies have suggested that large amounts of Na^+^ are stored in extra‐renal tissues, particularly the skin and muscles.^7^, ^8^, ^9^, ^10^ Mechanistically, excessive Na^+^ in bone, cartilage and skin is stored by non‐osmotic binding with polyanionic proteoglycans, generating a hypertonic environment in local tissue and acting as an osmotically inactive Na^+^ reservoir.^11^, ^12^, ^13^, ^14^ Moreover, tissue sodium storage is linked to certain diseases. Increased skin or muscle Na^+^ storage can be observed in patients with multiple sclerosis (MS),^15^ refractory hypertension,^10^ lipedema,^16^ systemic sclerosis^17^ or end‐stage renal disease.^18^, ^19^ Tissue Na^+^ storage may reflect the activity and progression of some diseases, such as systemic lupus erythematosus (SLE), MS and psoriasis.^20^, ^21^, ^22^, ^23^, ^24^ Of note, Na^+^ tends to accumulate in inflammatory and infection sites. In subtotal‐nephrectomized mice (5/6Nx) fed an HSD, sodium storage in the abdominal wall tissues was increased,^25^ and the total sodium concentration of patients with triple‐negative breast cancer was two to threefold higher in the tumour than in normal tissue.^26^

This dynamic and tightly regulated sodium homeostasis contributes to critical physiological functions in the body.^27^ Specifically, sodium is the major osmotically effective cation that keeps the plasma volume within normal limits and maintains suitable cell volume necessary for cellular survival and function.^28^, ^29^, ^30^ The resting membrane potential of cells largely depends on the transmembrane sodium concentration gradient, whereas the generation of action potential is mediated by a brief sodium influx.^28^, ^31^ Sodium also participates in various metabolic reactions. Data from humans showed that severe sodium deprivation can negatively affect glucose metabolism.^32^ Moreover, an adequate sodium intake is required for mammalian survival and development.^33^, ^34^ The growth of protoplasm, fat and bone was retarded in sodium‐deprived rats,^35^ while gestational sodium restriction can impair foetal growth and nervous system development.^36^, ^37^

## 3 THE EFFECT OF HIGH SALT ON IMMUNE CELLS

**FIGURE 1:**
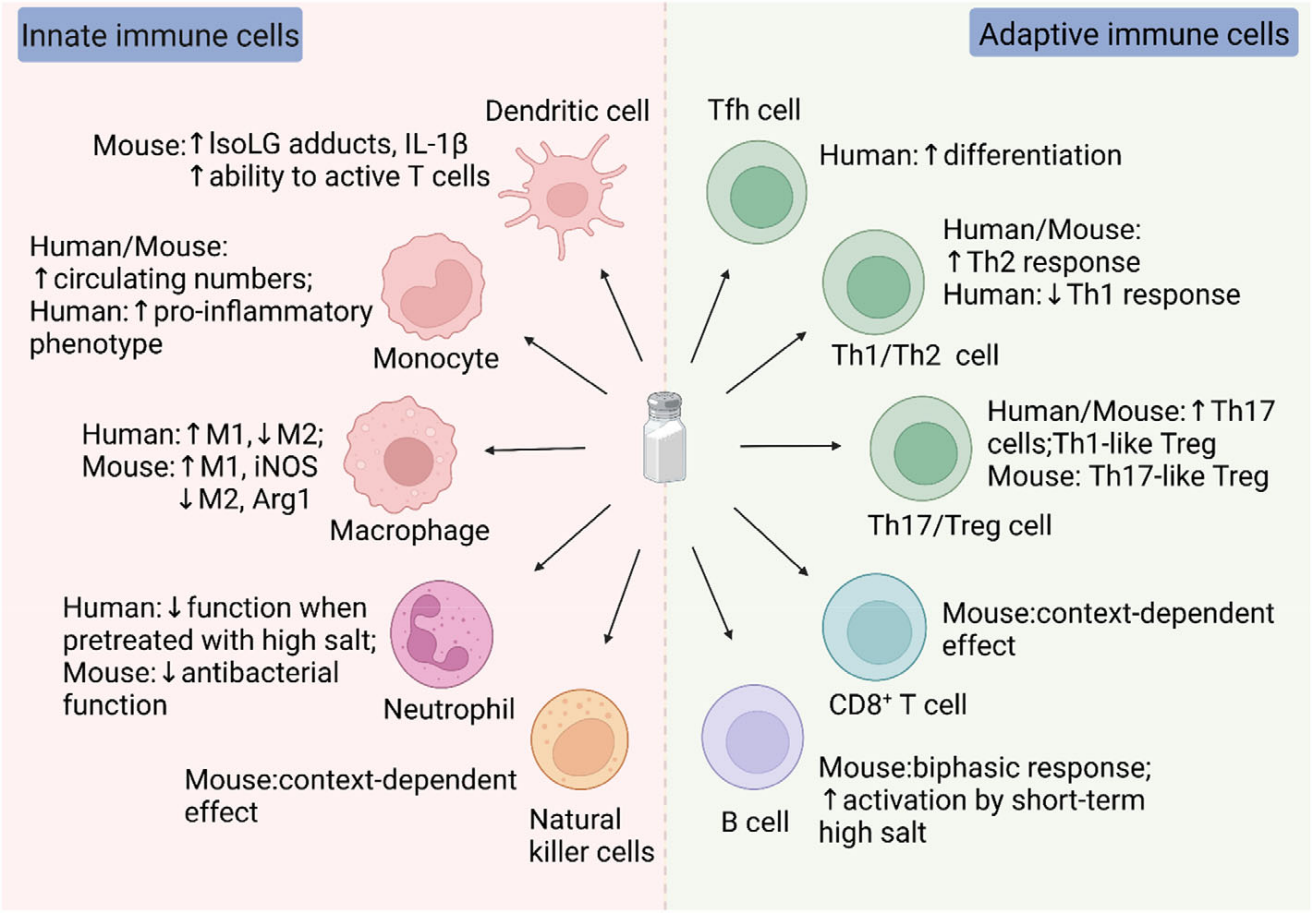
The effect of high salt on innate and adaptive immune cells. High salt enhances the formation of IsoLG‐protein adducts in murine dendritic cells, along with increased IL‐1β secretion and subsequent activation of T cells. An HSD increases circulating monocytes in mice and humans, and high salt can induce pro‐inflammatory human monocytes. Moreover, high salt induces M1 macrophages, Th17 cells and Th2 cells, while suppressing M2 macrophages, Treg cells and Th1 cells. The differentiation of human Tfh cells is increased by high salt. The sequence of high salt exposure and stimulation affects the outcome of human neutrophil activation, whereas the antibacterial functionality of murine neutrophils is suppressed by high salt. The high salt effects on murine NK cells and CD8^+^ T cells are context‐dependent, while murine B cells respond to high salt in a biphasic manner.

Immune cells comprise innate and adaptive immune cells. Innate immune cells include granulocytes, natural killer (NK) cells, macrophages, monocytes and dendritic cells (DCs). Adaptive immune cells include T lymphocytes and B lymphocytes, which express T cell receptor (TCR) and B cell receptor (BCR), respectively.^38^ Extensive studies have suggested the immunomodulatory function of salt, and we particularly focus on the effect of high salt on the differentiation, activation and function of immune cells in microenvironments. Furthermore, considering the difference between mouse and human immune cells,^39^ the high salt effects on immune cells showed discrepancies among species (Figure 1).

### 3.1 The effect of high salt on immune cell proliferation and death

In humans and rodents, plasma Na^+^ concentration is approximately 140 millimolar (mM), while Na^+^ concentrations in interstitium and lymphoid tissue range between 160 and 250 mM.^40^, ^41^ Based on these findings, most studies enrich the media with additional 20–100 mM NaCl to investigate the effect of high salt on immune cells.^42^, ^43^, ^44^, ^45^ Importantly, adding 40 mM NaCl can simulate the Na^+^ concentrations found in the skin of high‐salt‐diet rodents.^46^ These clinically relevant high salt can trigger osmoprotective responses in immune cells and affect cellular homeostasis.

Studies have demonstrated that immune cells rely on the nuclear factor of activated T cells‐5 (NFAT5 or TonEBP) to adapt to hypertonic stress.^47^ The guanine nucleotide exchange factor (GEF) Brx and p38 mitogen‐activated protein kinase (MAPK) can activate NFAT5 and mediate the osmoprotective response in immune cells.^48^, ^49^ In terms of immune cell proliferation, NFAT5 can induce osmoprotective responses that enable T lymphocytes to resume cyclin expression.^50^ Furthermore, CD24 was identified as an NFAT5‐regulated gene involved in the adaptation of murine proliferating T cells to high salt.^51^ Additionally, the exposure conditions and cell lines are critical. Human and murine T cell proliferation was suppressed at additional NaCl above 40 mM, but increasing NaCl below 40 mM promoted T cell proliferation.^51^, ^52^, ^53^, ^54^, ^55^ The augmented T cell proliferation is related to p38/MAPK activation,^52^ enhanced monocyte function^53^ and altered macrophage function.^45^, ^56^ Moreover, adding 40 mM NaCl can inhibit B cell proliferation.^57^

In addition, high NaCl can damage cytoskeleton, increase DNA breaks, inhibit translation and cause cell cycle arrest.^58^, ^59^, ^60^ Osmoprotective mechanisms enable cells to survive and function. Failure to accommodate leads to cell death.^58^ Partial loss of NFAT5 function reportedly led to impaired lymphocyte growth and function.^40^, ^61^ Additionally, salt exposure conditions and cell lines can influence the outcome of adaptation. In PBMCs, additional NaCl below 80 mM had no significant effect on cell apoptosis, whereas severe cell death was observed at 800 mM.^62^ Adding 100 mM NaCl for 24 h potentiated murine macrophage apoptosis, along with inhibited protein kinase B (PKB or Akt) activation.^63^, ^64^ Moreover, adding 40 mM NaCl had little impact on the growth or apoptosis of CD4^+^ T cells, but increasing concentrations further led to cell death.^43^, ^65^

### 3.2 The effect of high salt on innate immune cells

#### 3.2.1 Neutrophils

Neutrophils, a type of polymorphonuclear leukocyte, are the major effectors of acute inflammation.^66^ High‐salt treatment after human neutrophil activation can increase superoxide production and elastase release.^67^, ^68^ In contrast, high salt pretreatment can suppress diverse human neutrophil functions such as degranulation,^69^ superoxide production,^70^, ^71^ phagocytosis,^72^ adhesion^73^, ^74^ and migration.^42^, ^75^ These effects are related to adenosine triphosphate (ATP) release, p38/MAPK activation and cytoskeletal remodelling.^76^, ^77^, ^78^, ^79^ Moreover, NaCl above 209 mM can significantly suppress neutrophil extracellular trap (NET) formation while promoting apoptosis in human neutrophils.^80^ Consistently, high NaCl can impaire the antimicrobial capacities of both human and murine neutrophils. In human neutrophils, the reduced bactericidal activity was due to the decreased reactive oxygen species (ROS) production.^81^ Furthermore, an HSD in mice can increase the urea accumulation in the renal medulla and elevate the glucocorticoid level in the blood, resulting in the suppressed neutrophil antibacterial functionality. Healthy humans who accepted an HSD also showed hyperglucocorticoidism and inhibited neutrophil function.^82^ In summary, the sequence of high‐salt exposure and stimulation critically affects the outcome of human neutrophil activation, whereas high salt can suppress the antibacterial functionality of murine neutrophils.

#### 3.2.2 Natural killer cells

NK cell is a prototypical member of Group 1 innate lymphoid cells (ILC1) that can produce interferon‐γ (IFN‐γ).^83^, ^84^ A study demonstrated that HSD suppressed the proliferation, activation and function of NK cells in mice.^85^ Mice fed an HSD displayed decreased NK cells in the spleen and lungs, while NK cell maturation in the spleen and bone marrow was blocked. Mechanistically, HSD downregulated CD122 expression in NK cells via ROS signalling and thus reduced the responsiveness to interleukin (IL)‐15 in NK cells and inhibited their function.^85^ However, Rizvi et al.^86^ found that an HSD in mice enhanced NK cell function in tumour immunity by decreasing PD‐1 expression and increasing IFN‐γ and serum hippurate. These two distinct conclusions indicated the importance of the disease context in the high salt effect on murine NK cells.

#### 3.2.3 Mononuclear phagocyte family

Macrophages, DCs and monocytes form a family of mononuclear myeloid cells that are specialized in antigen presentation.^87^ These three myeloid cells also belong to the mononuclear phagocyte system (MPS), a concept encompassing progenitors, macrophages, DCs and monocytes.^88^ Tissue‐resident macrophages can be derived from embryonic progenitors or they can be of monocytic origin under inflammatory conditions, the relative proportion of which depends on the tissue.^89^, ^90^ DCs and monocytes originate from haematopoietic stem cells, but they have distinct precursors.^88^ Moreover, DCs comprise conventional DCs (cDCs) and plasmacytoid DCs (pDCs).^91^ In this review, we considered cDCs and not pDCs. Monocytes demonstrate plasticity and can be differentiated into macrophages and dendritic‐like cells.^92^

##### Macrophages

Macrophage activation phenotypes have been extended to a spectrum model with two extremes, M1 and M2 macrophages.^93^ M1 macrophages are polarized by inflammatory stimuli, mediating the immune response to bacteria and intracellular pathogens. M2 macrophages are anti‐inflammatory macrophages, existing in settings such as helminth immunity, asthma and allergy.^94^, ^95^

###### Murine macrophages

**FIGURE 2:**
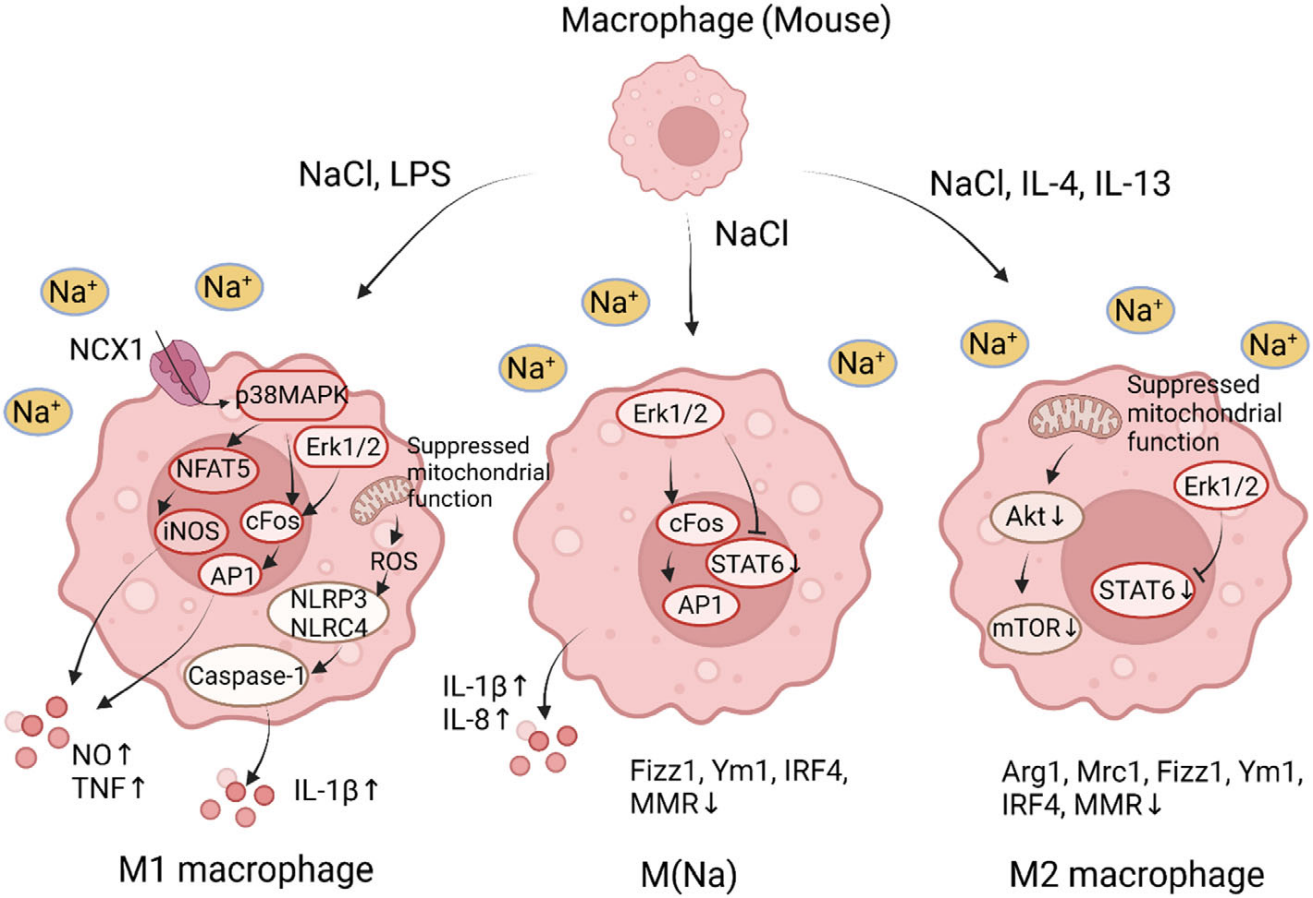
High salt induces a pro‐inflammatory profile in murine macrophages. NCX1 expressed on M1 macrophages can sense extracellular Na^+^, and high salt increases NO and TNF production by p38/MAPK‐dependent NFAT5 activation and downstream iNOS upregulation. Moreover, high salt activates NLRP3 and NLRC4 inflammasomes via mitochondrial ROS, thereby increasing IL‐1β in a caspase‐1‐dependent manner. Additionally, high salt induces a pro‐inflammatory profile in M1 and M(Na) macrophages through the p38/cFos/AP1 and Erk1/2/cFos/AP1 pathways, whereas the Erk1/2/STAT6 pathway mediates the salt‐driven suppression of M2 and M(Na) macrophages. In M2 macrophages, high salt downregulates the expression of Arg1, mannose receptor, C type 1 (Mrc1), inflammatory zone 1 (Fizz1), chitinase‐like 3 (Chil3 or Ym1), interferon regulatory factor 4 (IRF4) and macrophage mannose receptor (MMR). Furthermore, high salt suppresses mitochondrial metabolism and AKT/mTOR signalling in M2 macrophages. The mitochondrial function of M1 macrophages is also suppressed by high salt.

Generally, high salt potentiates the activation and function of M1 macrophages, while suppressing the activation and function of M2 macrophages (Figure 2).^45^, ^56^, ^96^, ^97^ First, high salt can induce p38/MAPK‐dependent NFAT5 activation in M1 macrophages, resulting in inducible nitric oxide synthase (iNOS or NOS2)‐dependent nitric oxide (NO) production and tumour necrosis factor (TNF) secretion.^41^ These responses boosted the function of macrophages, facilitating anti‐leishmanial control and regulating salt‐induced hypertension.^41^, ^46^ Additionally, the Na^+^/Ca^2+^ exchanger 1 (NCX1) expressed on murine macrophages can sense Na^+^ and contribute to NFAT5 accumulation.^98^, ^99^ Second, high salt can activate caspase‐1 and trigger IL‐1β release in macrophages. These responses required the activation of the nucleotide‐binding domain and leucine‐rich repeat pyrin domain 3 (NLRP3) and the nucleotide‐binding domain and leucine‐rich repeat caspase recruitment domain 4 (NLRC4) inflammasomes via mitochondrial ROS. These salt‐activated inflammasomes can act as innate sensing components and promote T helper (Th)17 response.^44^ Third, the p38/cFos/activator protein 1 (AP1) and extracellular signal‐regulated kinase (Erk)1/2/cFos/AP1 pathways mediated the salt‐driven pro‐inflammatory profile in M1 macrophages, while the Erk1/2/signal transducer and activator of transcription (STAT)6 pathway mediated the salt‐induced suppression of M2 macrophages.^97^ Furthermore, high NaCl can activate macrophages without extra activators, leading to a novel activation state named M(Na).^97^ Last, high salt can inhibit the mitochondrial respiration in macrophages, resulting in the improved bactericidal function of M1 macrophages and suppressed function of M2 macrophages.^45^, ^56^, ^99^ The inhibited M2 macrophage activation by high salt is associated with blunted Akt/mechanistic target of rapamycin signalling.^56^

Apart from the direct activation of pro‐inflammatory macrophages, high salt can induce macrophage migration. In vitro, macrophages can migrate towards NaCl in a dose‐dependent manner.^100^ In subtotal‐nephrectomized mice (5/6Nx), an HSD increased macrophage infiltration in the peritoneal wall, heart and para‐aortic tissues.^25^ The mechanisms involved the enhanced expression of NFAT5‐dependent monocyte chemotactic protein‐1 (MCP‐1) in mesothelial cells and cardiomyocytes.^25^

###### Human macrophages

High salt can induce M(Na) in human macrophages, characterized by enhanced pro‐inflammatory and suppressed anti‐inflammatory gene expression.^97^ Salt‐activated M1 macrophages with increased pro‐inflammatory cytokine production were also observed.^45^, ^97^ Moreover, high salt inhibited the mitochondrial respiration in M1 and M2 macrophages, resulting in the suppressed function of M2 macrophages.^99^ However, studies have identified the discrepancies between mouse and human macrophages. For instance, iNOS and arginase‐1 (Arg1), two enzymes important for murine macrophage arginine metabolism, might not be functional in human macrophages.^39^, ^101^ Thus, the investigations in murine macrophages cannot be simply extrapolated to human macrophages, and the high salt effect on human macrophages needs to be further explored.

##### Monocytes

Two studies in healthy subjects have revealed a positive association between salt intake levels and monocyte numbers.^102^, ^103^ Data showed that minimal changes in plasma sodium concentration caused by HSD can transiently inhibit the mitochondrial function of human circulating monocytes.^99^ A recent study found that high salt can drive human monocytes to a DC‐like phenotype, characterized by the formation of isolevuglandin (IsoLG) adducts, expression of CD83 and increased production of IL‐1β. These responses induced subsequent T‐cell activation with increased IL‐17A production.^104^ Consistently, monocytes from humans with high skin sodium exhibited enhanced IsoLG adduct accumulation and CD83 expression.^104^ Other studies also observed pro‐inflammatory human monocytes induced by high salt in vitro or by an HSD.^45^, ^105^ In addition, a long‐term HSD remarkably increased the circulating monocytes in mice by driving monocyte mobilization from bone marrow.^106^ Taken together, an HSD can increase circulating monocytes in mice and humans, and high salt can induce pro‐inflammatory human monocytes.

##### Dendritic cells

Current evidence has revealed the impact of high salt on murine DCs. High salt impeded the cross‐priming capacity of murine DCs in a toll/IL1 receptor domain‐containing adapter‐inducing interferon‐beta (TRIF)‐dependent manner.^107^ Moreover, murine DCs treated with high salt acquired an M2‐like signature.^108^ Nevertheless, the maturation, antigen presentation and inflammatory cytokine expression of murine DCs were reportedly enhanced by high salt.^107^, ^109^, ^110^ Specifically, elevated sodium enters DCs through amiloride‐sensitive channels and consequently increases the formation of IsoLG adducts and the production of IL‐1β. These salt‐activated DCs can promote the secretion of IL‐17A and IFN‐γ in T cells.^110^ Data in mice fed an HSD corroborated these findings.^111^ In addition, an HSD in SLE mice can activate DCs through the p38/MAPK‐STAT1 pathway.^112^ Tubbs et al.^113^ found that the increased cytokine production in murine DCs upon high NaCl treatment was mediated by p38/MAPK, serum and glucocorticoid‐inducible kinase 1 (SGK1) and downstream toll‐like receptor 4 (TLR4). Collectively, these findings suggested that high salt can activate murine DCs and increase their production of inflammatory cytokines.

### 3.3 The effect of high salt on adaptive immune cells

#### 3.3.1 CD4 ^+^ T cells

CD4^+^ T cells are central to the adaptive immune response against pathogens.^114^ Naïve CD4^+^ T cells can differentiate into distinct Th cell subsets, including Th1, Th2, Th17, (regulatory T) Treg and follicular helper T (Tfh) cells.^115^ Th1 cells, which produce IFN‐γ, are vital for cellular immunity. Th2 cells, essential for immunoglobulin E (IgE) production, play a major role in allergic and helminth responses.^116^ Th17 cells participate in mucosal immunity against pathogens and contribute to autoimmune disorders.^117^ Pro‐inflammatory and anti‐inflammatory Th17 phenotypes have been described.^118^, ^119^, ^120^ Treg cells are critical to maintaining immune tolerance, and can be categorized into thymus‐derived Treg (tTreg), peripherally derived Treg (pTreg) and in vitro‐induced Treg (iTreg) cells.^121^, ^122^ Tfh cells are crucial for IgG‐mediated humoral responses and are required to develop germinal centre responses.^123^, ^124^ Based on the heterogeneity and plasticity of Th cells,^125^ high salt exerts different effects on Th cell subsets.

##### Th17 cells

**FIGURE 3:**
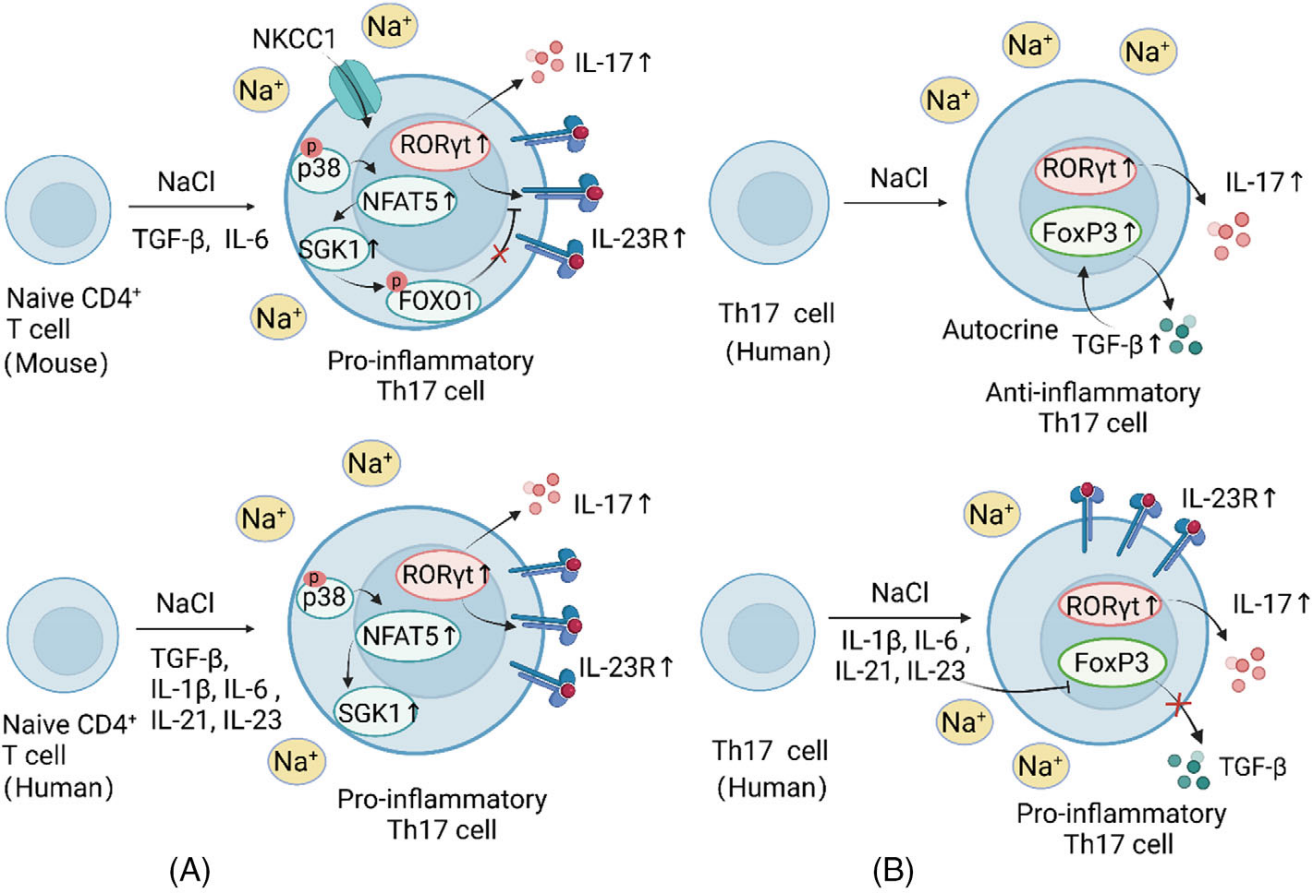
The context‐dependent effect of high salt on Th17 cells. (A) In the presence of Th17‐polarizing cytokines, high salt boosts the induction of pathogenic murine and human Th17 cells. These responses are related to p38/MAPK, NFAT5 and SGK1 activation. (B) In the absence of Th17‐polarizing cytokines, high salt induces anti‐inflammatory human Th17 cells by a significant upregulation of FoxP3 and the autocrine of TGF‐β. However, additional pro‐inflammatory cytokines can block TGF‐β secretion, and therefore, human Th17 cells exhibit a pro‐inflammatory phenotype. Similarly, high salt shows context‐dependent effect on the pathogenicity of murine Th17 cells, but the autocrine TGF‐β production was below the detection level.

High salt can induce distinct phenotypes of Th17 cells in different cellular contexts, and Th17‐polarizing cytokines, such as transforming growth factor‐β (TGF‐β) and IL‐6, strongly influence this process (Figure 3). Moreover, high salt intake can induce pro‐inflammatory Th17 cells by affecting gut microbiota and their metabolites. Notably, these high salt effects on Th17 cells appear similar between mice and humans.

In 2013, two seminal papers showed that high salt can induce human and murine naïve CD4^+^ T cells to differentiate into pathogenic Th17 cells in the presence of Th17‐polarizing cytokines.^43^, ^126^ In vitro, high salt induced a highly pathogenic and stable phenotype of Th17 cells, characterized by increased expression of IL‐17A and IL‐23R. The mechanisms involved the activation of p38/MAPK, NFAT5 and SGK1.^43^ Specifically, SGK1 can stabilize this salt‐induced pathogenic Th17 cell phenotype by deactivating mouse forkhead box protein O (FOXO)1, a repressor of RORγt‐mediated IL‐23R expression.^126^ In vivo, an HSD exacerbated experimental autoimmune encephalomyelitis (EAE), along with increased Th17 cells in the gut‐associated lymphoid tissue and the central nervous system (CNS) of mice. These responses can be abrogated by either p38/MAPK inhibitor or SGK1 deficiency.^43^, ^126^ In addition, Na^+^‐K^+^‐2Cl^−^ cotransporter 1 may participate in sensing extracellular NaCl in murine Th17 cells and mediate the salt‐induced increase in SGK1 and IL‐23R.^127^

In contrast, Matthias et al.^128^ proposed a dual context‐dependent effect of high NaCl on CD4^+^ T cells. High NaCl without additional polarizing cytokines endowed human Th17 cells with a stable, pathogen‐specific and anti‐inflammatory phenotype, characterized by a significant upregulation of FoxP3 and a moderate increase in IL‐17A. This salt‐induced Th17 phenotype was regulated by p38/MAPK, NFAT5 and SGK1.^128^ However, Th17‐polarizing cytokines suppressed the autocrine secretion of TGF‐β in human Th17 cells, which was important for FoxP3 upregulation induced by high salt, and thus switched the Th17 phenotype from anti‐inflammatory to pro‐inflammatory. Similarly, TGF‐β governed the induction of pro‐ versus anti‐inflammatory murine Th17 cells by high salt in vitro and in an EAE mouse model.^128^ Cumulatively, the obscuration caused by Th17‐polarizing cytokines in the high‐salt‐induced differentiation of T cells may explain the divergent outcomes compared to previous reports.

In addition, the high salt effect on Th17 cells can be mediated by gut microbiota and their metabolites.^129^ Indeed, an HSD in mice significantly reduced several intestinal bacteria, particularly the *Lactobacillus murinus*. Studies have indicated that increased salt consumption results in the depletion of *L. murinus* and thereby induces pro‐inflammatory Th17 cells in mice.^130^, ^131^ Mechanistically, *Lactobacilli* can metabolize tryptophan to indole metabolites. Faecal indoles, confirmed to be an inhibitory factor of Th17 differentiation, were reportedly decreased by HSD.^130^ Further investigations suggested that oral gavage of *L. murinus* or *Lactobacillus reuteri* in mice restored the decrease of faecal indoles caused by HSD and blunted high‐salt‐induced Th17 activation. Consistently, a high salt challenge on healthy volunteers increased the peripheral blood Th17 cells, accompanied by reduced intestinal *Lactobacillus* spp. survival.^130^ In summary, these studies linked HSD to the gut‐immune axis and suggested that HSD can induce pro‐inflammatory Th17 cells by decreasing gut *Lactobacillus* spp. in mice and humans.

##### Treg cells

**FIGURE 4:**
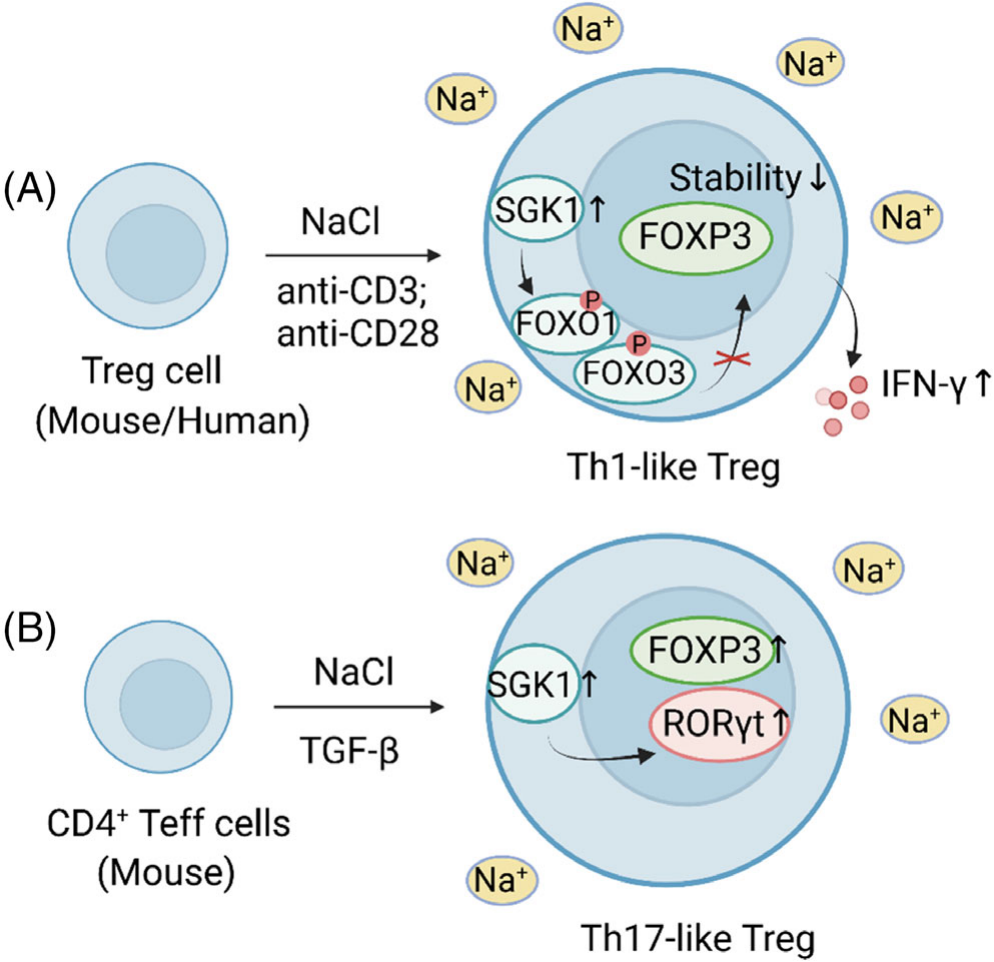
High salt induces pro‐inflammatory Treg cells. (A) High salt enables murine and human Treg cells to acquire a Th1‐like phenotype. High salt enhances the phosphorylation of FOXO1/FOXO3 by activating SGK1, followed by decreased Foxp3 stability and increased IFN‐γ secretion. (B) High salt can induce Th17‐like Treg cells. High salt can enhance TGF‐β‐mediated Treg cell induction while facilitating the co‐expression of RORγt

High salt enables murine Treg cells to acquire an SGK1‐dependent, pro‐inflammatory phenotype, which has proved to be Th1‐like^132^ or Th17‐like^133^ in different reports, leading to inhibited suppressive function of Treg cells. By contrast, a salt‐induced Th1‐like phenotype has been observed in human Treg cells (Figure 4).

On the one hand, high NaCl impaired the suppressive capacity of murine and human Treg cells in vitro and in vivo, leading to a Th1‐type phenotype with increased SGK1‐dependent IFN‐γ secretion.^132^ Specifically, FOXO1, a downstream target of SGK1, can stabilize the Foxp3 locus alongside FOXO3. Treg cells exposed to high salt displayed enhanced phosphorylation of FOXO1/FOXO3, and the decreased Foxp3 stability may be responsible for Treg suppression.^132^ On the other hand, Yang et al.^133^ found a Th17‐like phenotype of murine Treg cells with preserved suppressive function upon high salt treatment. These salt‐induced RORγt^+^Foxp3^+^ Treg cells were associated with SGK1 activation and were not significant producers of IL‐17A.^133^ Consistently, an HSD decreased the Treg proliferation and proportion in a murine transplantation model.^55^ Although it has been corroborated that high NaCl can promote RORγt expression in murine tTreg cells, whether high salt affects murine iTreg cells is controversial.^133^, ^134^ Data also suggested that high salt had little impact on the development and function of human iTreg cells.^134^

##### Other T helper cells

High salt can induce human Th2 but suppress Th1 cell responses on multiple regulatory levels.^65^, ^135^ IL‐4 and IL‐13, the Th2 signature cytokines, were enhanced in human naïve and memory T cells under high NaCl conditions, whereas the production of IFN‐γ was decreased. These effects were dependent on NFAT5 and SGK1.^65^ Consistently, an HSD augmented Th2 responses in food allergy mice,^136^ while the high‐salt formulation of Al(OH)_3_ enhanced the ovalbumin (OVA)‐induced Th2 response in mice.^109^ In addition, high NaCl can significantly increase the polarization of human Tfh cells.^62^ Mechanistically, high NaCl enhanced the gene expression of ten‐eleven translocation (TET)2 and TET3 in human CD4^+^ T cells, along with upregulated expression of sialophorin (spn), and then induced the DNA hypomethylation, ultimately contributing to enhanced Tfh cell differentiation. These findings provide a potential epigenetic mechanism for high‐salt‐induced autoimmunity.^62^

#### 3.3.2 CD8 ^+^ T cells

CD8^+^ T cells play a vital role in immunity to intracellular pathogens and tumours.^137^ HSD increased the renal infiltration of CD8^+^ T lymphocytes in mice.^138^, ^139^ In an E.G7‐OVA tumour mouse model, OVA/Al/high salt formulation displayed an enhanced antitumor effect via CD8^+^ cytotoxic T lymphocytes‐mediated immunity.^109^ By contrast, Popovic et al.^107^ found that high NaCl inhibited the DC‐dependent activation of murine CD8^+^ T cells in a TRIF‐dependent manner.

#### 3.3.3 B cells

B lymphocytes are immune cells that express clonally diverse cell surface immunoglobulin receptors.^140^ High salt might activate murine splenic B cells and increase immunoglobulin production via the Brx/p38/MAPK/NFAT5 pathway.^49^ Additionally, a short‐term increase in NaCl concentration fostered murine B cell activation and differentiation, whereas chronic exposure to high NaCl dampened the differentiation of plasmablasts. Mechanistically, murine B cells treated with high NaCl showed inhibited p38/MAPK pathway activity and delayed NFAT5 response.^57^

## 4 THE EFFECT OF HIGH‐SALT DIET ON IMMUNE‐REGULATED DISEASES

Given the high salt effects on immune cells and the excessive sodium storage in local tissues caused by an HSD,^46^ extensive studies have demonstrated the impact of an HSD on various immune‐regulated disorders in mouse models (Table 1) and in humans (Table 2).

**TABLE 1:** The effect of high‐salt diet on immune‐regulated diseases in mouse models

Disease	Effect	Responses	References
Hypertension	Increased	↑MPS cells	46
		↑DCs	110
		↑Th17 cells and IL‐17	130
		↓*Lactobacillus* spp.	130
Actively induced EAE	Exacerbated	↑Th17 cells	130
		↓*Lactobacillus* spp.	130
		↑Pro‐inflammatory myeloid cells	45
		↑Blood–brain barrier permeability	141
Spontaneous EAE	Suppressed	↓Blood–brain barrier permeability	142
Experimental colitis	Exacerbated	↑Macrophages	143, 144, 145
		↑Th17 cells and ILC3	131, 146, 147
		↓Treg cells	132
		↓*Lactobacillus* spp. and butyrate	131
		↑Intestinal fibroblasts	148
Systemic lupus erythematosus	Exacerbated	↑Th1 and Th17 cells	149
		↑Tfh cells	62
		↑DCs	112
Collagen‐induced arthritis	Exacerbated	↑Th17 cells and IL‐17	150
	No significant influence	/	151
K/BxN serum transfer‐induced arthritis	No significant influence	/	151
Cancer	Exacerbated	↑Th17 cells and IL‐17	152
		↑*Helicobacter pylori* infection	153
	Inhibited	↓MDSCs	154, 155
		↑T cells	154
		↑*Bifidobacterium* and NK cross‐talk	86
Infection	Exacerbated	↓Neutrophils	82
	Prevented	↑M1 and ↓M2 macrophages	41, 97, 156, 157
Ischemic stroke	Aggravated	↑Blood–brain barrier disruption	158
		↑M1 and ↓M2 macrophages	159, 160
Cognitive disorders	Impaired	↑Th17 cells and IL‐17	161
		↑M1 microglia	162
Wound healing	Delayed	↓M2 macrophages	56
Acute renal failure	Aggravated	↑Th17 cells	163, 164
Transplantation rejection	Accelerated	↓Treg cells	55, 132
Osteopenia	Increased	↑Th17 cells and ↓Treg cells	165

↑: increased; ↓: decreased.

**TABLE 2:** The effect of high‐salt diet on immune‐regulated diseases in humans

Disease	Effect	Design of the Study	Salt intake assessment	References
Hypertension	Increased	Pilot clinical trial	2‐Week high‐salt diet	130
		Meta‐analysis	24‐h urine collection/sodium manipulation	166
Cardiovascular disease	Increased risk	Meta‐analysis	Multiple 24‐h urine collection	167
Multiple sclerosis	Increased disease activity	Cohort	Spot urine collection	168
	No association	Cohort	Spot urine collection	169
		Cohort	Semiquantitative food frequency questionnaire (FFQ)	170
		Case–control	Block Kids Food Screener (NutritionQuest)	171
		Case–control	Block Kids Food Screener	172
Ulcerative colitis or Crohn's disease	No association	Nested case–control	Semiquantitative FFQ	173
Systemic lupus erythematosus	Positive association	Clinical trial	5‐Week dietary regimen	174
Rheumatoid arthritis	Positive association	Nested case–control	Semiquantitative FFQ	175
		Case–case	Food Frequency Questionnaires	176
		Cross‐sectional and case–control	Semiquantitative FFQ	177
		Cross‐sectional	Spot urine collection (urine Na/K ratio)	178
		Case–control	24‐h urine collection and FFQ	179
		Clinical trial	5‐Week dietary regimen	174
Gastric cancer	Increased risk	Meta‐analysis	FFQ	180
Oesophageal cancer	Increased risk	Meta‐analysis	Validated questionnaires	181
Lung, testicular and bladder cancer	Increased risk	Case–control	FFQ	182
Renal cell cancer	Increased risk	Cohort	FFQ	183
Pancreatic cancer	Increased risk	Case–control	Questionnaire	184
Infection	Increased risk	Clinical trial	1‐Week high‐salt diet	82
Ischemic stroke	Positive association	Case–control	Spot urine collection	158
	Positive association risk of death; no association risk of onset	Meta‐analysis	Questionnaire/24‐ h urinary sodium excretion/overnight urine sodium/24‐h dietary recall/a self‐monitoring device	185
Cognitive disorders	Positive association	Meta‐analysis	24‐h urine collection/FFQ/food diaries	186
Renal transplant and post‐transplant hypertension	Positive association	Clinical trial	Strict sodium diet	187, 188
		Cross‐sectional	24‐h urine collection	189
		Comparative	24‐h urine collection	190
	No association	Comparative	24‐h urine collection	191
Osteoporosis	Increased risk	Meta‐analysis	Spot or 24‐h urine collection/FFQ/24‐h dietary recalls	192

### 4.1 Hypertension and associated cardiovascular disease

It is well recognized that dietary salt intake has a direct causal relationship with blood pressure and can increase the risk of cardiovascular events and death.^166^, ^167^, ^193^ Studies have demonstrated that an HSD can contribute to hypertension via the immune system. First, an HSD in mice can prime hypertension through DC‐dependent T cell activation.^110^, ^111^ A study on human monocytes implied a similar mechanism.^104^ Second, an HSD can induce the generation of Th17 cells by reducing *Lactobacillus* species, contributing to hypertension in mice and humans.^130^ Last, salt‐driven alterations in short‐chain fatty acids (SCFAs), a subset of fatty acids generated by gut microbiota, were observed in hypertensives^194^ and mice with salt‐sensitive hypertension.^195^ Given the immunomodulatory functions of SCFAs,^196^ salt‐induced hypertension might be mediated by SCFAs. In contrast, in response to skin Na^+^ accumulation caused by HSD, interstitial MPS cells secrete NFAT5‐dependent vascular endothelial growth factor‐C (VEGF‐C), which increases the hyperplasia of lymph capillaries and provides a buffering mechanism for salt‐driven hypertension.^46^, ^197^, ^198^

### 4.2 Multiple sclerosis

MS is a chronic autoimmune demyelinating disease of the CNS.^199^ EAE, the model of MS, can be induced by active or passive immunization. It can also be developed from opticospinal EAE (OSE) spontaneously.^200^ Data showed that an HSD can aggravate actively induced EAE. First, exacerbated EAE by an HSD showed enhanced CNS infiltration and peripherally induced pathogenic Th17 cells.^43^, ^126^ Another study highlighted the role of gut microbiota in this process, as supplementation with *L. murinus* or *L. reuteri* blunted salt‐induced pathogenic Th17 cells and ameliorated EAE exacerbation.^130^ Second, EAE mice fed an HSD showed augmented macrophage infiltration in the CNS, and enhanced pro‐inflammatory cytokine production in myeloid cells.^45^ Last, high salt intake can exacerbate EAE by increasing blood–brain barrier (BBB) permeability.^141^ This effect might be related to the decreased tight junction (TJ) proteins in endothelial cells.^158^, ^201^ However, another study reported that an HSD suppressed spontaneous EAE by upregulating serum corticosterone and tightening BBB.^142^ The discrepancy between induced and spontaneous EAE may be the consequence of altered BBB properties by pertussis toxin used in the active immunization.

Data from humans suggested that the high salt effect on MS is controversial. A cohort study reported that high salt intake was associated with increased disease activity in patients with MS.^168^ Conversely, four other human studies found no association between HSD and MS progression.^169^, ^170^, ^171^, ^172^ However, these clinical studies measured dietary salt intake by spot urine collections or food questionnaires, which may lead to invalid results,^202^, ^203^ and more accurate measurement methods for salt intake are needed in follow‐up studies.

### 4.3 Intestinal bowel disease

IBD, encompassing ulcerative colitis and Crohn's disease, is a chronic relapsing inflammatory disorder of the gastrointestinal tract.^204^, ^205^ Data showed that an HSD can exacerbate DSS‐ and TNBS‐induced colitis, leading to increased mortality in mice.^113^, ^146^ This exacerbation of colitis is associated with the p38/MAPK‐dependent production of pro‐inflammatory cytokines by intestinal mononuclear cells.^143^, ^144^ Further investigations corroborated the participation of CD4^+^ T cells and macrophages.^145^ Depleting macrophages reduced the severity of DSS‐induced colitis promoted by high salt intake.^144^ Furthermore, an HSD exacerbated TNBS‐induced colitis by enhancing the intestinal Th17 response.^146^ The salt‐driven reduction in *Lactobacillus* and butyrate levels might mediate this salt‐induced Th17 response.^131^ In addition, Type 3 innate lymphoid cells ILC3, a kind of IL‐17‐producing cell increased in the colon of high‐salt‐diet mice, may participate in the salt‐driven aggravation of experimental colitis.^147^ Moreover, an HSD can aggravate experimental colitis by blocking the suppressive function of Treg cells.^132^ Last, data suggested that high dietary salt can promote intestinal fibrosis in TNBS‐induced colitis by activating intestinal fibroblasts.^148^ Nevertheless, a nested case–control study in women did not find an association between dietary sodium and the risk of ulcerative colitis or Crohn's disease.^173^ More investigations in humans are warranted to clarify the role of HSD in IBD.

### 4.4 Systemic lupus erythematosus and rheumatoid arthritis

SLE is an autoimmune, connective‐tissue disorder that involves multiple systems.^206^ Lupus nephritis is one of the most severe organ manifestations in SLE.^207^ Data showed that an HSD accelerated lupus progression and increased the mortality in MRL/lpr mice, a mouse model of SLE.^62^, ^149^ In MRL/lpr mice fed an HSD, the ratio of Th17/Treg was significantly increased,^149^ and a higher proportion of Tfh cells was observed in the spleen. Given the pathogenic role of Tfh cells in lupus, an HSD might accelerate the SLE progression by inducing Tfh cell differentiation.^62^ Additionally, an HSD accelerated the progression of murine lupus by activating DCs through the p38/MAPK‐STAT1 pathway.^112^

RA is a chronic inflammatory joint disease that can cause cartilage, bone damage and disability.^208^ Collagen‐induced arthritis (CIA) and K/BxN serum transfer‐induced arthritis (STIA) are mouse models of RA. CIA depends on adaptive and innate immunity, while STIA predominantly mimics the innate effector phase.^151^ CIA mice fed an HSD showed more severe arthritis, higher Th17 cell proportion in splenocytes and increased IL‐17 expression in synovium and intestine.^150^ Sehnert et al.^151^ did not observe aggravated CIA or STIA in high‐salt‐diet mice, but they found that a low‐salt diet ameliorated the severity of CIA and STIA. Consistently, a study enrolled RA and SLE patients demonstrated that a restricted dietary salt intake can dampen the pro‐inflammatory response in patients with autoimmune diseases.^174^ Moreover, high salt consumption among smokers was reportedly associated with increased RA risk.^175^, ^176^ By contrast, another study confirmed the association between high sodium intake and RA, particularly in nonsmokers.^177^ Increased sodium excretion was also observed in patients with RA,^179^ and further investigation suggested a correlation between RA disease activity and urinary Na/K ratio.^178^

### 4.5 Cancer

It is known that the Na^+^ concentration is raised in solid tumours and can affect cell metabolism and immune function. Thus, it seems promising that dietary salt can influence the development of tumours and has the potential to be administered in cancer therapy.^209^

Compelling evidence suggests that an HSD can suppress the progression of tumours. High salt intake suppressed the tumour growth and lung metastasis in a breast cancer murine model,^210^ while an HSD reduced ETBF‐promoted colon carcinogenesis by decreasing the IL‐17A and iNOS expression.^211^ Moreover, an HSD can inhibit the growth of transplanted melanoma, mammary cancer and Lewis lung carcinoma in mice by reducing myeloid‐derived suppressor cells (MDSCs).^154^, ^155^ Specifically, monocytic MDSCs (M‐MDSCs) differentiated into M1 macrophages, while granulocytic MDSCs (PMN‐MDSCs) converted to a pro‐inflammatory phenotype, therefore reactivating the antitumor actions of T cells. An HSD also enhanced the antitumor activation of PD‐1 inhibitors.^154^ Additionally, an HSD induced NK cell‐mediated tumour immunity by augmenting the intratumor localization of *Bifidobacterium*.^86^

However, given that high salt intake is a potent inducer of pro‐inflammatory states, the adverse effect of HSD on cancer has also been reported.^212^ In vitro, high salt can synergize with IL‐17 to enhance the proliferation, treatment resistance and Warburg‐like metabolism of breast cancer cells.^213^, ^214^, ^215^ In mice, an HSD accelerated the development and lung metastasis of breast cancer.^152^, ^216^ Data suggested that the IL‐17F produced by salt‐induced Th17 cells activated the MAPK signalling in breast cancer cells.^152^ In humans, high salt intake is a risk factor for lung, testicular, bladder,^182^ renal cell,^183^ pancreatic,^184^ oesophageal^181^ and gastric cancer.^180^ Particularly, an HSD can promote *Helicobacter pylori* infection, gastric mucosa damage, hypergastrinemia and cell proliferation, therefore contributing to gastric carcinogenesis.^153^ These double‐sided effects indicated that the high salt effect on tumours might change with different tissues and phases of tumours.

### 4.6 Infections

An HSD might promote the elimination of *Escherichia coli* (*E. coli*) and *Leishmania major* (*L. major*) infection by increasing skin Na^+^ concentrations.^41^, ^46^, ^156^ The salt‐driven ameliorated cutaneous *L. major* infection is related to the enhanced iNOS expression in macrophages,^41^ while the salt‐augmented antibacterial activity against *E. coli* of macrophages hinges on the increased autophagy and autolysosomal targeting.^156^ Additionally, an HSD can protect mice from lethal vesicular stomatitis virus (VSV) infection through the macrophage activation via p38/MAPK/activating transcription factor 2ATF2/AP1 pathway.^157^ In murine models of acute lung injury induced by LPS, an HSD aggravated lung inflammation by activating macrophages.^97^ In contrast, excessive salt intake aggravated uropathogenic *E. coli*‐induced pyelonephritis and systemic *Listeria monocytogenes* infection in mice by suppressing the antibacterial function of neutrophils. Healthy volunteers who accepted an HSD also showed impaired neutrophil functions and might be more vulnerable to infection.^82^ These discrepancies indicated that the effect of HSD on infections might depend on the tissue sodium distributions and the organ‐specific responses.

### 4.7 Ischemic stroke

Ischemic stroke is a cerebrovascular disease that causes high mortality worldwide.^217^ It has been reported that HSD in mice can exacerbate ischemic stroke.^218^ An animal study linked HSD to ischemic brain damage.^219^ Mechanistically, an HSD in mice enhanced BBB disruption during ischemia via the p38/MAPK/SGK1 pathway, accompanied by downregulated TJ protein expression in endothelial cells.^158^ Additionally, an HSD induced pro‐inflammatory microglia by increasing aldose reductase (AR) protein expression via p38/MAPK and thus exacerbated ischemic stroke.^159^ HSD also decreased the expression of the phagocytic molecule triggering receptor expressed on myeloid cells 2 TREM2 and induced a pro‐inflammatory phenotype in macrophages, leading to the postponed recovery of stroke lesions.^160^ Moreover, high urinary sodium levels in humans were associated with large ischemic lesions.^158^ Data from humans also showed that high salt intake was associated with the risk of ischemic stroke death, but was not associated with the risk of ischemic stroke onset.^185^

### 4.8 Cognitive disorders

Cognitive disorders, common in psychiatric and neurological diseases, are a major societal burden.^220^ High salt intake has been reported to impair cognitive functions via the gut‐brain axis. HSD can induce gut Th17 responses and increase circulating IL‐17, which suppresses cerebral endothelial NO production, leading to cerebral hypoperfusion, neurovascular dysregulation and cognitive impairment in mice.^161^ Furthermore, an HSD caused cognitive dysfunction in mice by inducing an inflammatory environment and triggering apoptosis in the brain.^162^ Several human studies linked HSD to impaired cognitive function, while a clinical trial showed that a low‐salt intake might improve cognition.^186^ Nonetheless, most studies employed food questionnaires or diet records, and higher‐quality studies are needed.

### 4.9 Transplantation rejection

In a humanized xenogeneic graft‐versus‐host disease x‐GvHD murine model, an HSD blocked the immunosuppressive function of Treg cells and consequently worsened the severity and onset of transplantation rejection.^132^ Another study demonstrated that mice fed an HSD displayed accelerated cardiac allograft rejection, accompanied by decreased Treg cells.^55^ Furthermore, several human studies confirmed that dietary salt can influence the graft failure and mortality of renal transplant recipients by regulating blood pressure,^187^, ^188^, ^189^, ^190^ while another publication found no connection between dietary sodium and the prevalence or severity of post‐transplant hypertension.^191^

### 4.10 Osteoporosis

Studies in humans suggested that the calcium loss caused by excessive salt intake might increase the risk of osteoporosis.^192^ The enhanced bone loss and impaired bone‐microarchitecture in high‐salt‐diet mice are attributable to the increase in osteoclastogenic Th17 cells and the reduction in anti‐osteoclastogenic Treg cells caused by HSD.^165^, ^221^

### 4.11 Others

Wound healing was delayed in high‐salt‐diet mice owing to reduced M2 activation.^56^ Additionally, HSD in mice during recovery from acute renal failure accelerated the progress towards chronic kidney disease and interstitial fibrosis. These responses were associated with Th17 cell activation in the kidney.^163^, ^164^ Moreover, an HSD might exacerbate food allergy in mice,^136^ while sodium may participate in the progression of atopic dermatitis by regulating Th2 responses.^65^

## 5 CLINICAL APPLICATION OF HIGH SALT

The clinical applications of salt can date back to the middle ages, when salt was used as a treatment for toothaches, upset stomachs and so forth.^222^ Nowadays, high salt in clinical practice is primarily applied in the form of intravenous injection or nebulization. Hypertonic saline resuscitation can promote volume expansion, improve microcirculation and modulate immune responses in certain critically ill patients.^223^, ^224^ In addition, hypertonic saline has been adopted as an alternative to mannitol in patients with raised intracranial pressure.^225^ Nebulized hypertonic saline is already used in the treatment of cystic fibrosis,^226^ non‐cystic fibrosis bronchiectasis^227^ and viral bronchiolitis.^228^ Hypertonic saline inhalation in patients with cystic fibrosis can rehydrate the airway surface liquid, increase mucociliary clearance and improve lung function.^229^, ^230^ Most guidelines recommend the use of nebulized hypertonic saline in bronchiectasis therapy to facilitate airway clearance.^227^, ^231^ Nonetheless, studies about the application of nebulized hypertonic saline in patients with viral bronchiolitis demonstrated conflicting results.^228^ Additionally, hypertonic saline nasal irrigation might be helpful in patients with chronic rhinosinusitis.^232^ Furthermore, an increase in salt concentration in cancer vaccines can significantly change the physicochemical properties of the vaccine formulation and enhance its efficacy, showing invaluable potential in cancer therapy.^109^, ^233^ Salt‐activated CD4^+^ T cells that were derived from tumour‐bearing mice and injected into mice with breast cancer also elicited a strong anticancer response.^234^ As the effect of high salt on immune cells and immune‐regulated diseases has been studied in recent decades, further investigation on its feasibility and applicability in clinical therapy should be conducted.

## 6 CONCLUSION AND FUTURE DIRECTION

In recent decades, we have witnessed the immunomodulatory effect of high salt. High salt has a profound impact on the differentiation, activation and function of multiple immune cells. These alterations in immune cells are dependent on the cellular milieu and disease context. Additionally, an HSD can affect the sodium concentration of local tissues, dominantly induce a pro‐inflammatory profile in microenvironments and thereby modulate the development of various immune‐regulated diseases, suggesting salt as a target or potential agent in the immune therapy of different diseases. However, owing to the variable cellular context in local tissues and different types and phases of diseases, the high salt effect on humans is intricate and changeable. The exact impact of HSD on diseases such as cancer needs to be further illustrated. Although some of the current epidemiological evidence has indicated the association between HSD and several diseases, since the sodium measurement methods used in most studies failed to estimate the mean sodium intake of individuals accurately, these conclusions might not be credible and higher‐quality clinical studies are warranted to validate the effect of HSD on diseases in populations. Moreover, novel applications of high salt, such as enhancing the efficacy of cancer vaccines by increasing salt concentrations in vaccine formulations, should be further explored.

## AUTHOR CONTRIBUTIONS

Min Luo contributed to the conception and design of the review. The first draft of the manuscript was written by Xian Li. Xian Li and Aqu Alu created all the figures and tables. Yuquan Wei and Xiawei Wei critically revised the manuscript. All authors contributed to the article and approved the submitted version.

## CONFLICTS OF INTEREST

The authors declare that they have no conflicts of interest.

## Data Availability

Data sharing is not applicable to this article as no new data were created or analyzed in this study.
